# Altered functional network topology and connectivity in female nurses with shift work sleep disorder

**DOI:** 10.3389/fnsys.2025.1639981

**Published:** 2025-07-15

**Authors:** Hu-Cheng Yang, Si-Yu Gu, Shu-Fang Wang, Jian-Ping Liu, Shu Wang, Hai-Juan Chen, Li Chen, Chun-Mei Song, Qing-He Li, Zhen-Yu Dai, Ping-Lei Pan

**Affiliations:** ^1^Department of Radiology, The Yancheng School of Clinical Medicine of Nanjing Medical University, Yancheng, China; ^2^Department of Radiology, Binhai Maternal and Child Health Hospital, Yancheng, China; ^3^Department of Neurology, The Yancheng School of Clinical Medicine of Nanjing Medical University, Yancheng, China; ^4^Department of Nursing, The Yancheng School of Clinical Medicine of Nanjing Medical University, Yancheng, China; ^5^Intensive Care Unit, Affiliated Hospital 6 of Nantong University, Yancheng Third People’s Hospital, Yancheng, China

**Keywords:** shift work sleep disorder, nurses, functional connectivity, magnetic resonance imaging, network topology, graph theory

## Abstract

**Background:**

Shift work sleep disorder (SWSD) in nurses is highly prevalent and is increasingly recognized for its profound impact on human health. However, the brain functional network topology, which provides a comprehensive map of the brain’s information processing architecture, remains partially understood in nurses with SWSD.

**Methods:**

45 nurses with SWSD and 45 healthy controls (HCs) underwent a resting-state functional magnetic resonance imaging (rs-fMRI) scan. Graph theoretical analysis was used to investigate alterations in brain functional network topology. Functional network connectivity was further examined in nurses with SWSD relative to HCs. Correlations between network metrics and clinical sleep scores were also examined.

**Results:**

Compared to HCs, the SWSD group exhibited significantly lower global network metrics. Additionally, at the regional level, the SWSD group showed reduced nodal efficiency in specific regions, particularly within the visual processing areas and the caudate nucleus. Functional network connectivity analysis revealed a predominant pattern of weakened connectivity within the limbic network (LN), visual network (VN), default mode network (DMN), subcortical network (SN) and between the LN and SN in the SWSD group, although some inter-network connections were strengthened, predominantly the VN-ventral attention network (VAN), frontoparietal network (FPN)-VN, somatomotor network-VAN, and VN-DMN. Furthermore, poorer sleep quality correlated with reduced local efficiency in the visual cortex and insomnia severity was associated with weakened frontal connectivity.

**Conclusions:**

This study reveals significant alterations in brain functional network topology and predominantly weakened functional connectivity across multiple brain networks, despite some strengthened inter-network links. These neuroimaging changes correlated with clinical measures of sleep disturbance. Our findings highlight compromised brain network organization in SWSD, offering insights into its neural mechanisms and potential biomarkers.

## Introduction

Shift work, prevalent among healthcare workers like nurses, significantly disrupts the endogenous circadian rhythm and acts as a major risk factor for sleep disorders (Bostock and Mortimore, 2024; Abate et al., 2023). Shift work sleep disorder (SWSD) is particularly prevalent in this group, with reported rates reaching up to 48.5% (Li et al., 2021). This chronic circadian dysregulation leads to a state of persistent sleep deprivation, which may extend beyond workdays (Qayyum et al., 2024; Potter et al., 2016). This results in impaired daytime functioning, including significant cognitive deficits in attention and executive function, and an increased susceptibility to mood disorders such as anxiety and depression (Li et al., 2021; Kalmbach et al., 2015; Britten et al., 2021). These impairments consequently jeopardize the nurse well-being, the quality of patient care, and overall patient safety (Li et al., 2019; Lin et al., 2024). Although progress has been made in understanding the mechanisms underlying SWSD, key aspects remain unclear, limiting the development of effective early interventions.

With rapid advancements in neuroimaging technology, recent studies have indicated that SWSD in nurses is closely associated with alterations in brain function, such as abnormal activity in the default mode network (DMN) and attention-related circuits (Wu et al., 2021; Dong et al., 2024; Zhao et al., 2024; Belcher et al., 2015). Resting-state functional magnetic resonance imaging (rs-fMRI) is a key non-invasive technique used to investigate intrinsic brain dynamics by measuring blood-oxygen-level-dependent (BOLD) signals, thereby assessing spontaneous neural activity across brain regions at rest (Zhang et al., 2021; Yu et al., 2025). Functional connectivity (FC), which assesses the temporal correlation of neural signals between brain regions, is widely used to investigate intrinsic brain network interactions (Puvogel et al., 2022; Zhao et al., 2020). Previous rs-fMRI studies have identified local functional abnormalities and altered FC in specific brain regions in individuals with SWSD, and these alterations correlate with measures of sleep quality, cognitive function, anxiety, depression, and occupational burnout (Wu et al., 2021; Dong et al., 2024; Ye et al., 2022; Dong et al., 2024). However, these studies have often focused on isolated brain regions or limited functional connections, thus failing to elucidate the overall neural mechanisms of SWSD from a large-scale network perspective. Functional connectomics has recently emerged as a prominent field in neuroscience research (Patel and Bullmore, 2016). Integrating graph theoretical analysis with rs-fMRI data, this methodology maps large-scale brain functional networks, systematically quantifies FC patterns, and reveals their inherent topological attributes (Agziyart et al., 2024; Lv et al., 2024). SWSD is increasingly conceptualized as a brain network disorder, potentially arising from disruptions across widespread neural systems rather than from isolated deficits (Zhao et al., 2024). Understanding the brain’s functional connectome and its topological properties is crucial for elucidating the neurobiological mechanisms underlying SWSD.

To address this knowledge gap, we employed graph theory analysis of resting-state fMRI data to conduct a systematic investigation into the brain functional network topology of female nurses with SWSD. Our study was guided by three primary hypotheses: (1) that female nurses with SWSD would exhibit disrupted brain network organization, manifesting as reduced global and local efficiency compared to HCs; (2) that SWSD would be associated with altered functional connectivity, especially weakened connections within and between key networks like the DMN, visual network (VN), and limbic network (LN); and (3) that these neuroimaging-derived network metrics would correlate with clinical measures of sleep disturbance. By elucidating the neurobiological underpinnings of SWSD, this study aimed to advance our understanding of its pathophysiology and inform the development of novel strategies for prevention and intervention. A flowchart detailing the research process is presented in Supplementary Figure S1.

## Methods

### Participants

This study recruited female nurses from the Yancheng School of Clinical Medicine, Nanjing Medical University from May to July 2024. To minimize the acute effects of recent shift work and to capture the chronic neurobiological alterations associated with SWSD, all participants were scanned on a scheduled day off between 6:00 PM and 9:00 PM. Prior to the scan, participants were instructed to lie still in a supine position with their eyes closed, remain awake, and avoid systematic thinking, allowing their minds to wander freely. Foam padding was used to minimize head motion. The resting-state scan was part of a broader imaging protocol, and the scanning conditions were kept consistent for all participants. Inclusion criteria for the SWSD group (Buysse et al., 1989) were as follows: (1) female, aged 20–40 years (This specific age range was chosen to create a homogenous sample, minimizing confounding effects related to sex-based differences in brain function, as well as age-related neurodevelopmental or neurodegenerative changes); (2) engaged in continuous shift work for one year or more and currently maintaining this schedule; (3) right-handed; (4) Pittsburgh Sleep Quality Index (PSQI) score ≥ 5. Inclusion criteria for the HCs were as follows: (1) female, aged 20–40 years; (2) day-time working nurse; (3) right-handed; (4) PSQI score < 5. Exclusion criteria for all participants were as follows: (1) presence of endocrine, neurological, or psychiatric disorders or other primary diseases; (2) pregnancy or lactation; (3) history of drug dependence, current smoking, or alcohol abuse/dependence; (4) adverse reactions during scanning leading to termination of the experiment or contraindications to MRI scanning; (5) data collection failure during scanning or unclear images; (6) MRI images showing organic brain lesions; and (7) other serious physical illnesses. Based on these inclusion and exclusion criteria, 90 participants were ultimately selected and assigned to either the SWSD group (*n* = 45) or the HCs (*n* = 45). The two groups were matched for age and years of education. This study strictly adhered to the ethical principles of the Declaration of Helsinki, received approval from the Ethics Committee of the Yancheng School of Clinical Medicine of Nanjing Medical University (Approval No. 2024-82), and obtained informed consent from all participants.

The required sample size was determined by an *a priori* power analysis conducted in G*Power (version 3.1). The independent two-sample *t* test was used to determine the sample size of two groups. The parameters for the power analysis were set as follows: the effect size d = 0.8, *α* error probability = 0.05, power (1−*β* error probability) = 0.90. Thirty-four participants per group (SWSD group and HCs group) would be required to detect the hypothesized neuroimaging differences with sufficient statistical power. Our final sample size (*n* = 45 per group) comfortably exceeds this requirement.

Prior to MRI scanning, general demographic information and clinical data were collected, including age, years of education, Beck Anxiety Inventory (BAI) scores, Beck Depression Inventory-II (BDI-II) scores, PSQI scores, and Insomnia Severity Index (ISI) scores.

### MRI data acquisition

Rs-fMRI and structural 3D T1-weighted images were acquired using a 3.0 Tesla MRI scanner equipped with a 24-channel head coil (Discovery 750w, GE, United States) at the Yancheng School of Clinical Medicine of Nanjing Medical University (Imaging parameters are provided in the Supplementary materials).

### Rs-fMRI preprocessing

Resting-state fMRI preprocessing: The rs-fMRI data were preprocessed using SPM12 and Data Processing and Analysis for Brain Imaging (DPABI) implemented in MATLAB (R2018b) (Yan et al., 2016) (Preprocessing steps are provided in the Supplementary materials).

### Graph theory analyses

Graph theoretical analysis of brain network characteristics was performed using the GRETNA software (Wang et al., 2015). The entire brain was segmented into 90 network nodes using the AAL atlas. The AAL-90 atlas was selected for its wide application in brain network studies (Lv et al., 2024; Sun et al., 2023; Wang et al., 2014; Bernhardt et al., 2011), thus enhancing the comparability of our findings with existing literature. Subsequently, this matrix was converted into an undirected binarized form through sparsity thresholding applied over a range of network densities (5% ≤ sparsity ≤ 50%, in 0.01 intervals). The minimum sparsity was set to ensure that there were no isolated nodes in the network. The maximum sparsity was set to ensure that the small-world index was greater than 1.1 for all participants, balancing network inclusion and spurious connection avoidance. Global measures, including the clustering coefficient (C_p_), local efficiency (E_loc_), characteristic path length (L_p_), global efficiency (E_glob_), small-worldness (*σ*), normalized clustering coefficient (*γ*), and the normalized characteristic path length (*λ*), were computed alongside nodal measures, such as the nodal clustering coefficient (NCP), nodal efficiency (NE), nodal local efficiency (NLE), nodal degree centrality (DC), and nodal betweenness centrality (BC). For each network metric, the area under the curve (AUC) across the defined sparsity range was calculated for subsequent statistical comparisons, providing a summary measure independent of single threshold selection.

### Functional connectivity network analysis

FC networks were constructed using the graph theoretical network analysis toolbox (GRETNA) (Wang et al., 2015). First, the brain was parcellated into 90 regions using the Anatomical Automatic Labeling (AAL) atlas. Then, the mean time series was extracted for each of these 90 regions. Subsequently, a Pearson correlation coefficient matrix was generated by calculating the Pearson correlations between the average time series of all pairs of these 90 regions. Finally, Fisher’s *Z* transform was performed. In this way, a symmetric 90 × 90 network matrix was constructed for each subject from which a functional network was derived.

Differences in FC between brain regions were analyzed using the connection module of the GRETNA software. Group differences in functional connections were identified using a two-sample *t*-test, with false discovery rate (FDR) correction applied for multiple comparisons. Significant results were visualized using BrainNet Viewer (Xia et al., 2013).

### Statistical analysis

SPSS 27.0 software was used for statistical analysis. First, normality tests were conducted for age, years of education, PSQI scores, ISI scores, TIV, BAI scores, and BDI-II scores. For normally distributed measurement data, independent sample *t*-tests were used, while non-parametric tests were applied for skewed distribution data. A *p*-value < 0.05 was considered to indicate statistically significant between-group differences in demographic and clinical characteristics.

Two-sample *t*-tests were utilized to evaluate group differences in the seven global network metrics (*p* < 0.05) and the five regional nodal metrics (*p* < 0.05, FDR corrected), with age, years of education, TIV, BAI scores, and BDI-II scores considered as covariates. Subsequently, partial correlation analyses were conducted to explore associations between topological properties that showed significant group differences and clinical scale scores in nurses with SWSD, while controlling for age, years of education, TIV, BAI scores, and BDI-II scores. Statistical significance was set at *p* < 0.05.

## Results

### Demographic characteristics

Demographic and clinical characteristics of the HCs (*n* = 45) and the SWSD group (*n* = 45) are presented in Table 1. There were no significant differences between the groups in age (median [IQR]: 31.00 [22.50, 36.00] vs. 33.00 [29.50, 36.00], *p* = 0.116), sex (all female), years of education (median [IQR]: 16.00 [16.00, 16.00] vs. 16.00 [16.00, 16.00], *p* = 0.297), handedness (all right-handed), or TIV (mean ± SD: 1446.57 ± 110.60 vs. 1418.30 ± 124.60, *p* = 0.102). Compared to the HCs, the SWSD group exhibited significantly higher scores on the PSQI (median [IQR]: 8.00 [5.00, 11.00] vs. 5.00 [4.00, 8.00], *p* = 0.003), ISI (median [IQR]: 8.00 [5.00, 11.00] vs. 5.00 [4.00, 8.00], *p* = 0.019), BAI (median [IQR]: 24.00 [21.00, 27.00] vs. 24.00 [21.00, 27.00], *p* = 0.002), and BDI-II (median [IQR]: 9.00 [4.50, 17.00] vs. 5.00 [1.00, 8.00], *p* < 0.001).

**Table 1:** Demographic and clinical features of all participants.

Characteristics	HCs (*n* = 45)	SWSD (*n* = 45)	Statistic	*p* value
				SWSD vs. HCs
Age	31.00 (22.50, 36.00)	33.00 (29.50, 36.00)	Z = −1.570	0.116
Sex (M/F)	0/45	0/45	Z = 0.00	1.00
Education (years)	16.00 (16.00, 16.00)	16.00 (16.00, 16.00)	Z = −1.042	0.297
Handedness (R/L)	45/0	45/0	Z = 0.00	1.00
TIV	1446.57 ± 110.60	1418.30 ± 124.60	Z = −1.634	0.102
PSQI	5.0 (4.00, 8.00)	8.00 (5.00, 11.00)	Z = −3.015	**0.003**
ISI	5.0 (4.00, 8.00)	8.00 (5.00, 11.00)	Z = −2.341	**0.019**
BAI	24.00 (21.00, 27.00)	24.00 (21.00, 27.00)	Z = −3.143	**0.002**
BDI	5.00 (1.00, 8.00)	9.00 (4.50, 17.00)	Z = −3.307	**<0.001**

BAI, Beck Anxiety Inventory; BDI, Beck Depression Inventory; FC, functional connectivity; F, female; HCs, healthy controls; ISI, Insomnia Severity Index; L, left; M, male; PSQI, Pittsburgh Sleep Quality Index; R, right; SWSD, shift work sleep disorder; TIV, total intracranial volume. Bold values indicate statistical significance (*p* < 0.05).

### Global network metrics

**Figure 1:**
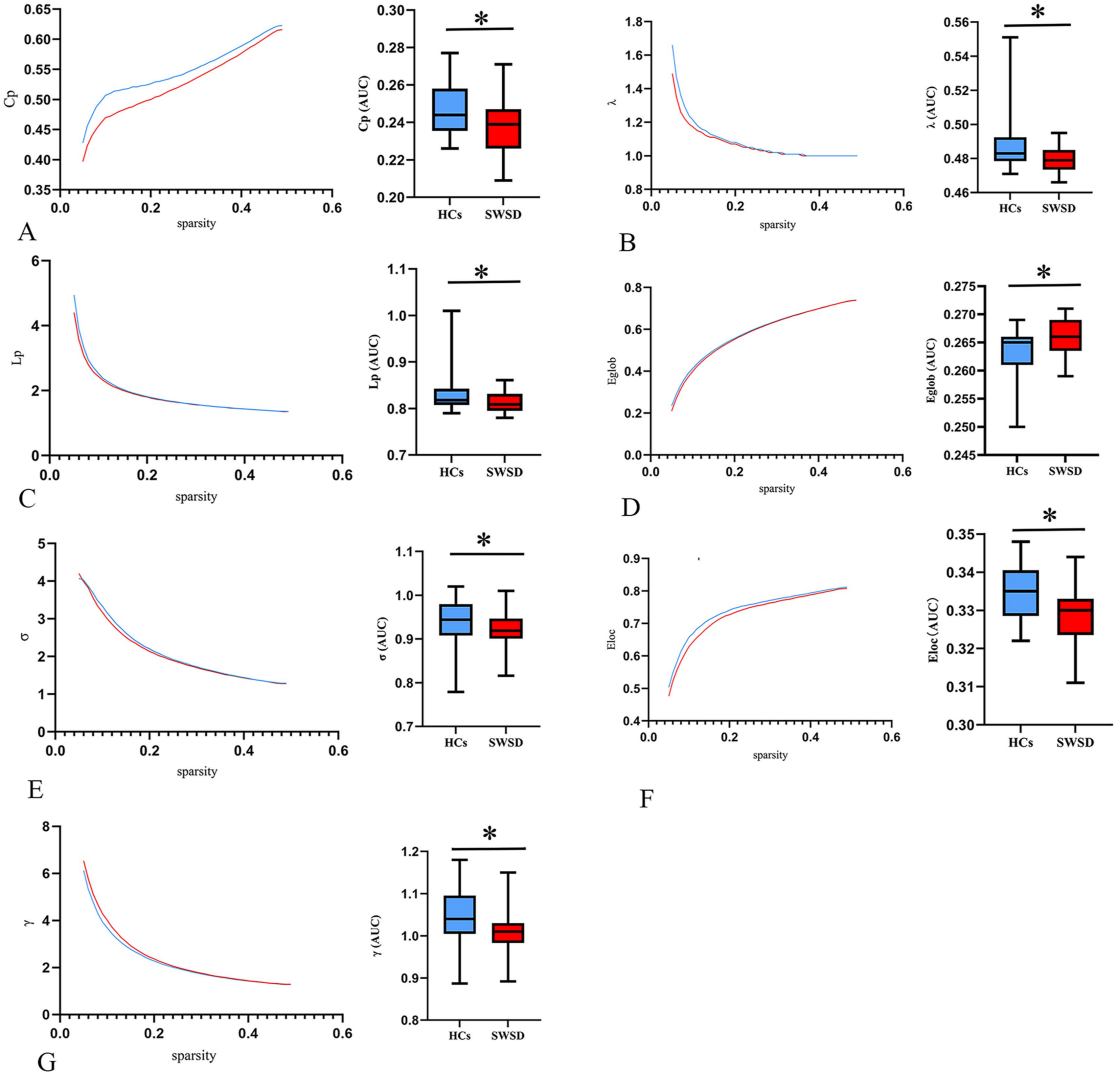
Alterations in global network metrics. Compared with HCs, the C_p_ **(A)**, *λ* **(B)**, L_p_ **(C)**, E_glob_ **(D)**, *σ* **(E)**, E_loc_ **(F)**, and *γ* **(G)** were significantly decreased in nurses with SWSD. SWSD, shift work sleep disorder; HCs, healthy controls; AUC, area under the curve; E_glob_, global efficiency; E_loc_, local efficiency; C_p_, clustering coefficient; L_p_, characteristic path length; σ, small worldness; λ, normalized characteristic path length; γ, normalized clustering coefficient (^*^p < 0.05).

Global network metrics for the SWSD group and HCs were calculated and compared, utilizing the AUC for each global property (Table 2). The results showed that, compared to HCs, the SWSD group exhibited significantly lower E_loc_ (0.329 ± 0.006 vs. 0.334 ± 0.007, Z = −3.418, *p* < 0.001), E_glob_ (0.266 ± 0.032 vs. 0.265 [0.261, 0.266], Z = −2.641, *p* = 0.0081), C_p_ (0.237 ± 0.135 vs. 0.247 ± 0.013, Z = −2.923, *p* = 0.003), L_p_ (0.811 ± 0.020 vs. 0.819 [0.808, 0.844], Z = −2.914, *p* = 0.004), *γ* (1.004 ± 0.041 vs. 1.049 ± 0.059, Z = −3.765, *p* < 0.001), *λ* (0.478 ± 0.074 vs. 0.483 [0.478, 0.492], Z = −3.165, *p* = 0.002), and *σ* (0.918 ± 0.034 vs. 0.944 [0.908, 0.979], Z = −2.587, *p* = 0.010) (all *p* < 0.05, FDR corrected; Figure 1).

**Table 2:** Group comparisons of AUC values of global network properties.

Metrics	HCs (*n* = 45)	SWSD (*n* = 45)	Statistic	*p* value
				SWSD vs. HCs
E_glob_	0.265 (0.261, 0.266)	0.266 ± 0.032	Z = −2.641	**0.0081**
E_loc_	0.334 ± 0.007	0.329 ± 0.006	Z = −3.418	<**0.001**
C_p_	0.247 ± 0.013	0.237 ± 0.135	Z = −2.923	**0.003**
L_p_	0.819 (0.808, 0.844)	0.811 ± 0.020	Z = −2.914	**0.004**
σ	0.944 (0.908, 0.979)	0.918 ± 0.034	Z = −2.587	**0.010**
λ	0.483 (0.478, 0.492)	0.478 ± 0.074	Z = −3.165	**0.002**
γ	1.049 ± 0.059	1.004 ± 0.041	Z = −3.765	<**0.001**

AUC, area under the curve; C_p_, clustering coefficient; E_glob_, global efficiency; E_loc_, local efficiency; HCs, healthy controls; L_p_, characteristic path length; SWSD, shift work sleep disorder; σ, small worldness; λ, normalized characteristic path length; γ, normalized clustering coefficient. Bold values indicate statistical significance (*p* < 0.05).

### Nodal network metrics

**Figure 2:**
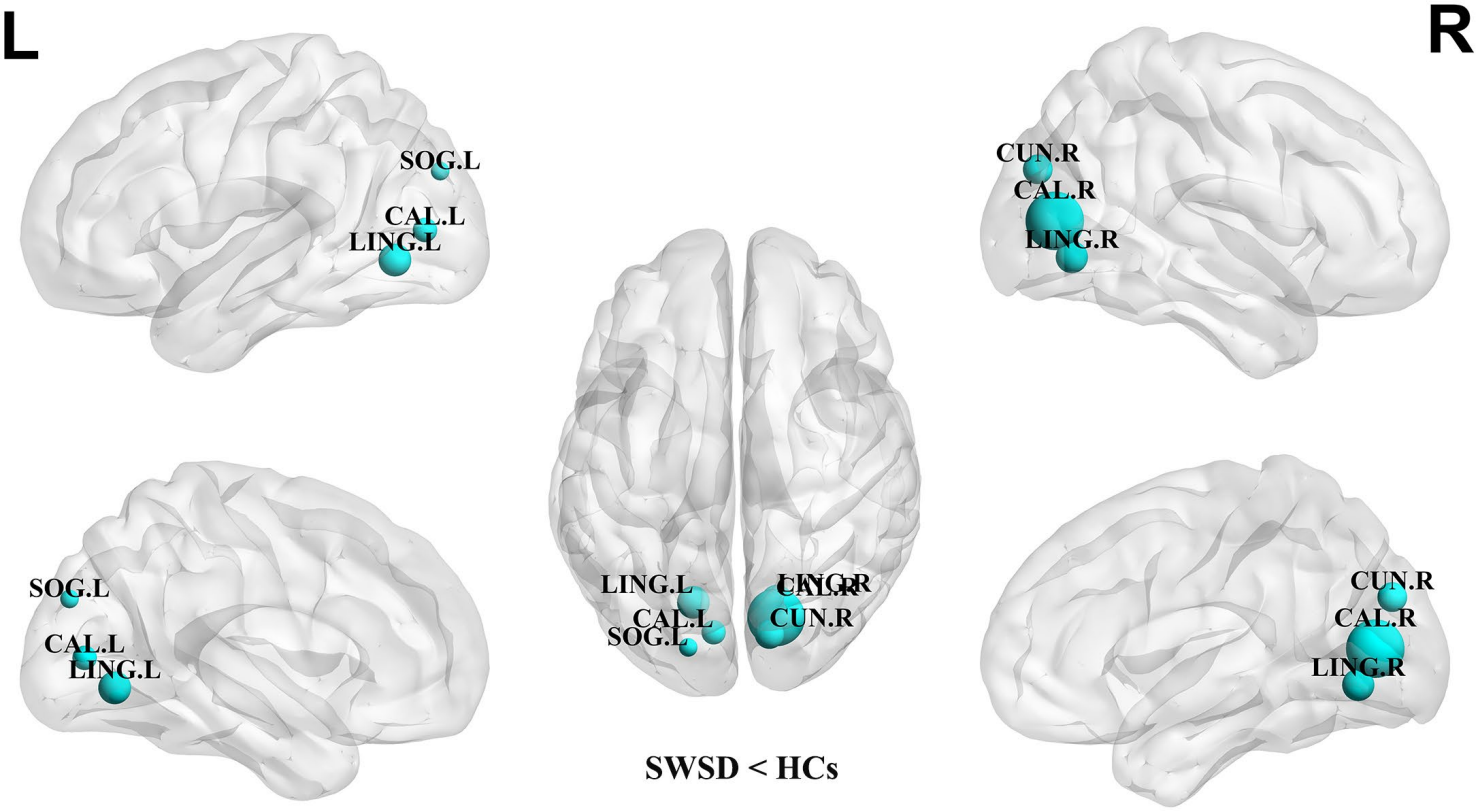
Nodes showing significant differences in NCP between nurses with SWSD and HCs. The blue circles represent a higher NCP in HCs than in nurses with SWSD (all *p* < 0.05, FDR corrected). NCP, nodal clustering coefficient; HCs, healthy controls; SWSD, shift work sleep disorder; CAL, calcarine cortex; LING, lingual gyrus; CUN, cuneus; SOG, superior occipital gyrus, FDR, false discovery rate; L, left; R, right.

**Figure 3:**
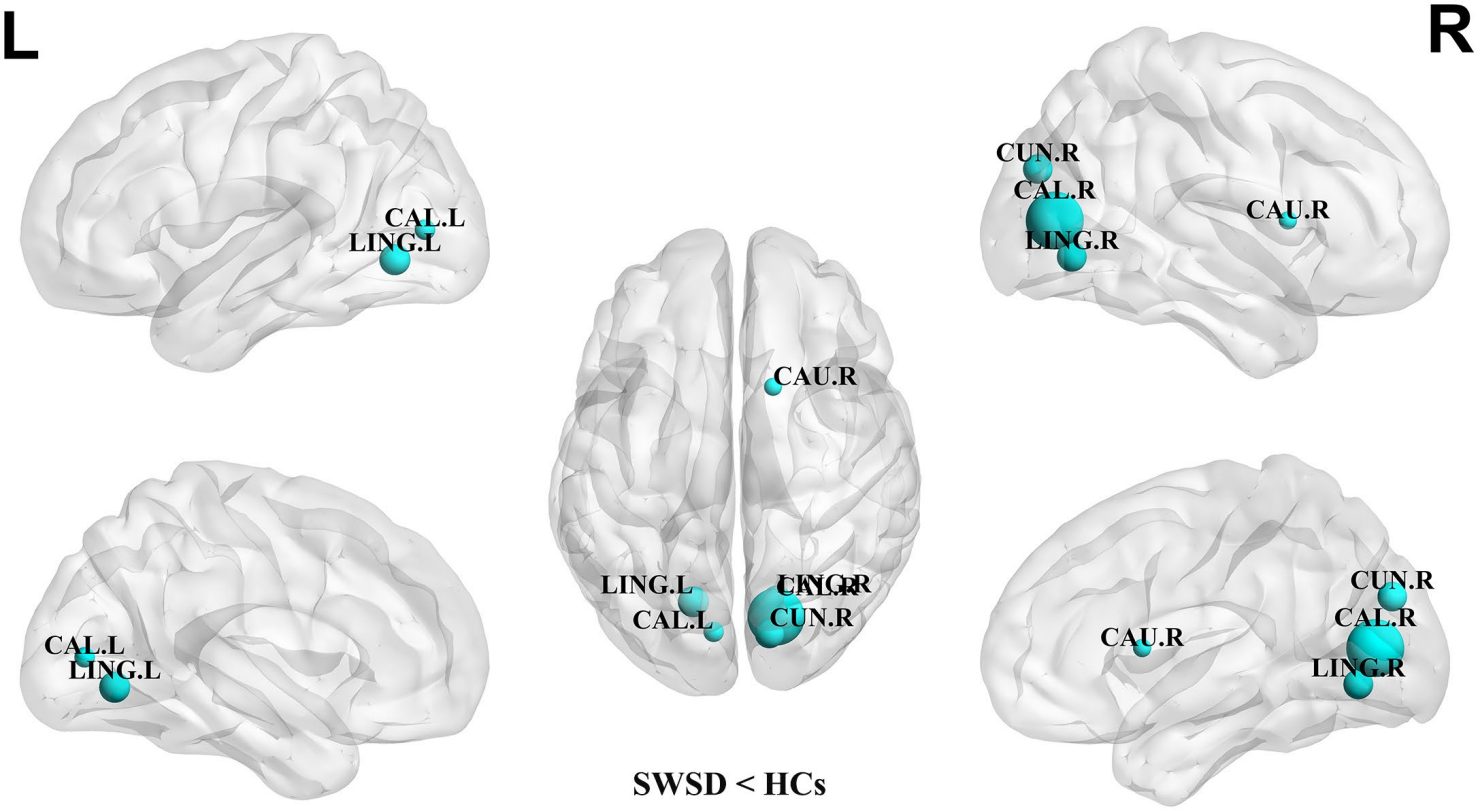
Nodes showing significant differences in NLE between nurses with SWSD and HCs. The blue circles represent a higher NLE in HCs than in nurses with SWSD (all *p* < 0.05, FDR corrected). NLE, nodal local efficiency; HCs, healthy controls; SWSD, shift work sleep disorder; CAL, calcarine cortex; LING, lingual gyrus; CUN, cuneus; CAU, caudate nucleus; FDR, false discovery rate; L, left; R, right.

Compared with the HCs, the NCP of the bilateral calcarine cortex, bilateral lingual gyrus, right cuneus and left superior occipital gyrus was significantly decreased in the SWSD group (all *p* < 0.05, FDR corrected) (Figure 2). Similarly, NLE values were also significantly reduced in the SWSD group, specifically within the bilateral calcarine cortex, bilateral lingual gyrus, right cuneus, and right caudate nucleus (all *p* < 0.05, FDR corrected) (Figure 3).

### Altered functional network connectivity

**Figure 4:**
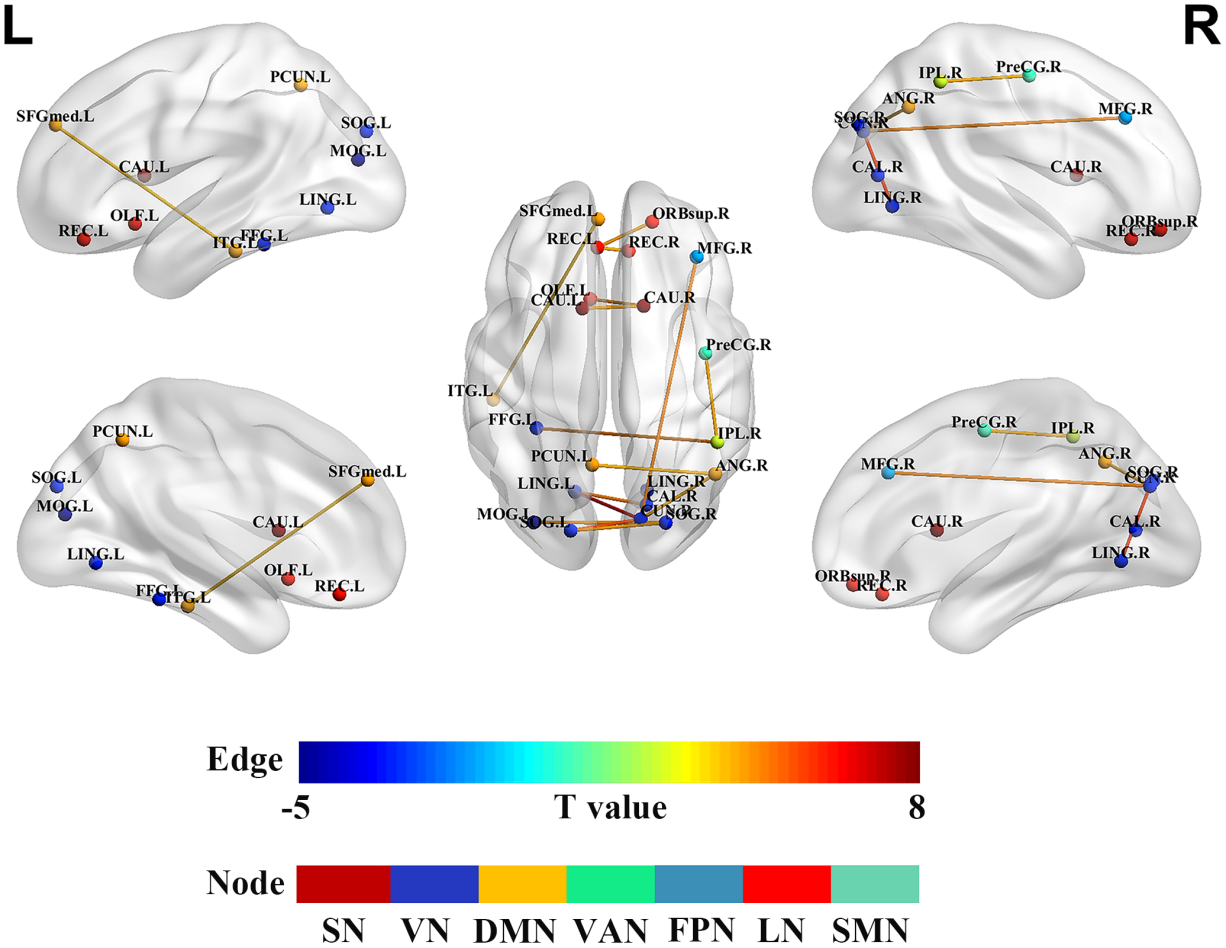
Alterations in brain functional network connectivity between nurses with SWSD and HCs. Nodes represent specific brain regions grouped by functional networks, including SMN, DMN, VN, VAN, FPN, SN, and LN. Edges indicate significant changes in functional connectivity between nurses with SWSD and HCs, with edge colors reflecting the direction and magnitude of *t*-values (all *p* < 0.05, FDR corrected) SWSD, shift work sleep disorder; CAL, calcarine cortex; CAU, caudate nucleus; LING, lingual gyrus; CUN, cuneus; SOG, superior occipital gyrus, FDR, false discovery rate; FC, functional connectivity; ANG, angular gyrus; MFG, middle frontal gyrus; MOG, middle occipital gyrus; OLF, olfactory cortex; REC, rectus gyrus; ORBsup, superior orbital frontal gyrus; FFG, fusiform gyrus; ITG, inferior temporal gyrus; SFGmed, medial superior frontal gyrus; PreCG, precentral gyrus; SMN, somatomotor network; DMN, default mode network; VN, visual network; VAN, ventral attention network; FPN, frontoparietal network; SN, subcortical network; LN, limbic network; L, left; R, right.

Compared to HCs, the SWSD group showed significantly altered FC (all *p* < 0.05, FDR corrected). A predominant pattern of reduced FC was observed across 13 connections involving 18 distinct brain regions. These reductions were evident within several key networks: the limbic network (LN) (between the right superior orbitofrontal gyrus and left rectus gyrus; between bilateral rectus gyrus), the visual network (VN) (between right calcarine cortex and left lingual gyrus; right cuneus and bilateral lingual gyrus; right cuneus and left superior occipital gyrus; bilateral superior occipital gyrus; right superior occipital gyrus and left middle occipital gyrus; left middle occipital gyrus and right fusiform gyrus), the default mode network (DMN) (between right angular gyrus and left precuneus; left medial superior frontal gyrus and left inferior temporal gyrus), and the subcortical network (SN) (between bilateral caudate nucleus). Reduced FC was also observed between the LN (left olfactory cortex) and SN (right caudate nucleus). In contrast, 4 connections involving 6 distinct brain regions exhibited significantly increased FC. These were primarily inter-network connections, including those between the frontoparietal network (FPN; right middle frontal gyrus) and VN (right cuneus), the somatomotor network (SMN; right precentral gyrus) and ventral attention network (VAN; right inferior parietal lobule), the VN (left fusiform gyrus) and VAN (right inferior parietal lobule), and the VN (right cuneus) and DMN (right angular gyrus) (*p < 0.05*, FDR corrected) (Table 3; Figure 4).

**Table 3:** Altered functional network connectivity.

Regions 1	Regions 2	*p*	*t*
ORBsup.R (LN)	REC.L (LN)	<0.01	−4.33
REC.L (LN)	REC.R (LN)	<0.01	−4.10
MFG.R (FPN)	CUN.R (VN)	<0.01	4.59
CAL.R (VN)	LING.L (VN)	<0.01	−4.67
CUN.R (VN)	LING.L (VN)	<0.01	−7.20
CUN.R (VN)	LING.R (VN)	<0.01	−5.60
CUN.R (VN)	SOG.L (VN)	<0.01	−5.68
SOG.L (VN)	SOG.R (VN)	<0.01	−4.27
SOG.R (VN)	MOG.L (VN)	<0.01	−4.48
MOG.L (VN)	FFG.R (VN)	<0.01	−3.88
PreCG.R (SMN)	IPL.R (VAN)	<0.01	4.18
FFG.L (VN)	IPL.R (VAN)	<0.01	4.63
CUN.R (VN)	ANG.R (DMN)	<0.01	4.07
ANG.R (DMN)	PCUN.L (DMN)	<0.01	−4.03
OLF.L (LN)	CAU.R (SN)	<0.01	−4.45
CAU.L (SN)	CAU.R (SN)	<0.01	−4.20
SFGmed.L (DMN)	ITG.L (DMN)	<0.01	−3.9

ANG, angular gyrus; CAL, calcarine cortex; CAU, caudate nucleus; CUN, cuneus; DMN, default mode network; FFG, fusiform gyrus; FPN, frontoparietal network; IPL, inferior parietal lobule; ITG, inferior temporal gyrus; LN, limbic network; LING, lingual gyrus; MFG, middle frontal gyrus; MOG, middle occipital gyrus; OLF, olfactory cortex; ORBsup, superior orbitofrontal gyrus; PCUN, precuneus; PreCG, precentral gyrus; REC, rectus gyrus; SFGmed, superior frontal gyrus, medial part; SMN, somatomotor network; SN, subcortical network; SOG, superior occipital gyrus; VN, visual network; VAN, ventral attention network.

### Correlation analysis

**Figure 5:**
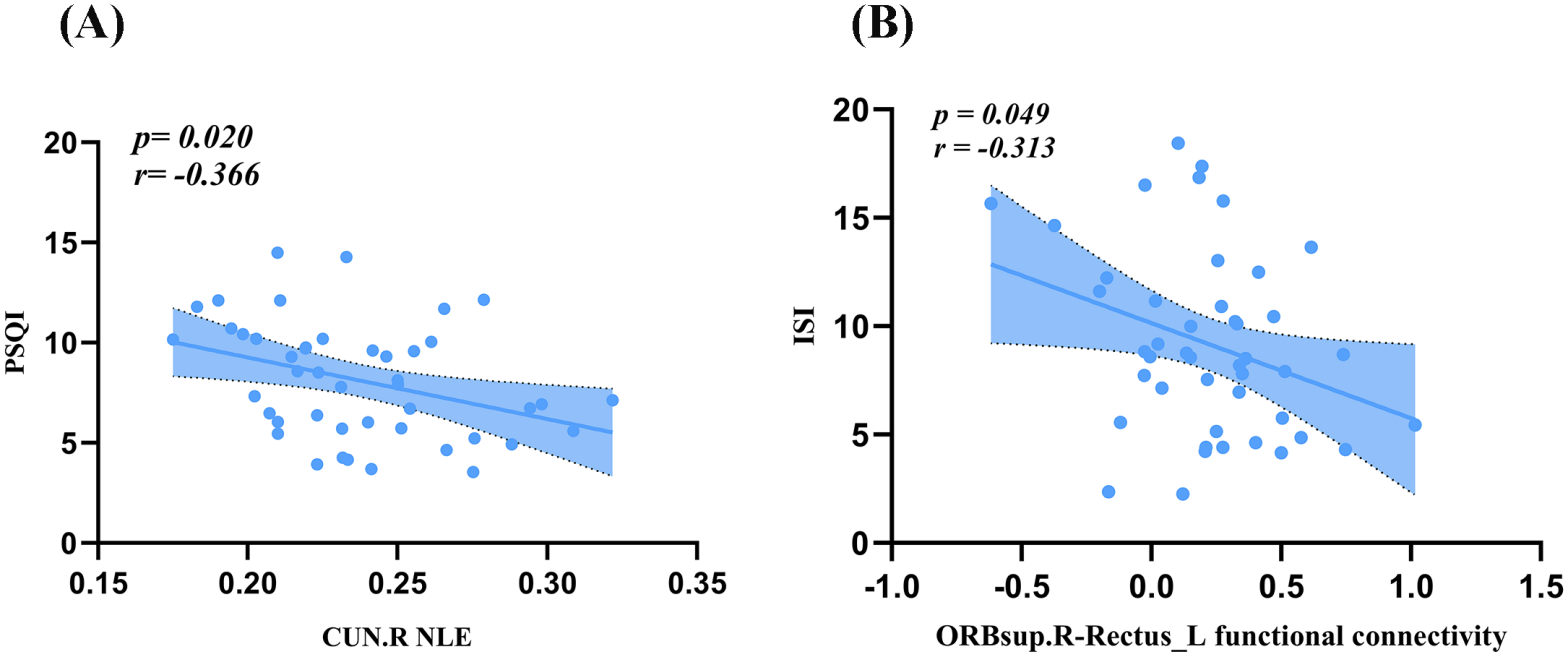
Correlation analysis. **(A)** PSQI scores were negatively correlated with NLE in the CUN.R. **(B)** ISI scores were negatively correlated with FC between the ORBsup.R and the Rectus.L. PSQI, Pittsburgh Sleep Quality Index; ISI, Insomnia Severity Index; FC, functional connectivity; ORBsup, superior orbitofrontal gyrus; CUN, cuneus; L, left; R, right.

As illustrated in Figure 5A, PSQI scores were negatively correlated with NLE in the right cuneus (*r* = −0.366, *p* = 0.020). ISI scores were negatively correlated with FC between the right superior orbital frontal gyrus and the left gyrus rectus (*r* = −0.313, *p* = 0.049, Figure 5B).

## Discussion

Using graph theoretical analysis, this study investigated alterations in brain functional network topology and subnetwork connectivity in nurses with SWSD relative to HCs. These network alterations were subsequently correlated with clinical sleep variables, specifically the PSQI and ISI. The principal findings were as follows: (1) global network metrics, including E_loc_, E_glob_, C_p_, L_p_, *λ*, *γ*, and *σ*, were significantly reduced in the SWSD group compared to HCs; (2) at the nodal level, significant decreases were observed in the SWSD group compared to HCs for NCP, specifically in the bilateral calcarine cortex, bilateral lingual gyrus, right cuneus, and left superior occipital gyrus, and for NLE, particularly within the bilateral calcarine cortex, bilateral lingual gyrus, right cuneus, and right caudate nucleus; (3) the functional network connectivity analysis revealed that the SWSD group exhibited significantly reduced FC both between and within multiple brain networks, mainly involving the VN, LN, DMN, and SN, alongside significantly increased FC predominantly in inter-network connections, namely VN-VAN, FPN-VN, SMN-VAN, and VN-DMN; and (4) PSQI scores showed a negative correlation with NLE in the right cuneus, and ISI scores were negatively correlated with FC between the right orbital superior frontal gyrus and the left gyrus rectus.

### Altered brain functional network topology in SWSD

The intricate structural and functional organization of the brain arises from the topological configurations of neuronal clusters (Wang et al., 2010; Zhu et al., 2018), which can be represented as interconnected nodes and edges using advanced imaging techniques and graph theory, thereby unveiling extensive dynamic interactions within the brain (Sporns et al., 2005). The significant reductions in global network metrics (E_loc_, E_glob_, C_p_, L_p_, λ, γ, and *σ*) observed in the SWSD group compared to HCs indicate a fundamental shift in the brain’s topological organization. This pattern of network inefficiency resonates with findings from studies on sleep deprivation and potentially interacts with underlying circadian influences on network topology (Qi et al., 2021; Farahani et al., 2019; Ning et al., 2022). However, such findings are nuanced by normal diurnal variations. For instance, Farahani et al. found that resting-state functional networks showed increased *σ*, assortativity, and synchronization in the evening versus the morning, suggesting more efficient network organization later in the wake period, possibly as morning sleep inertia effects are overcome (Farahani et al., 2021). This contrasts with our finding of reduced σ in nurses with SWSD, suggesting that the chronic circadian disruption inherent in this condition may override or pathologically alter normal diurnal network fluctuations, leading to a persistently less efficient network state. Although direct comparison is complex, the consistent theme across sleep deprivation, chronic insomnia disorders, poor sleep quality, and circadian studies is the vulnerability of the brain’s efficient topological organization to disruptions in sleep–wake regulation (Li et al., 2018; Chee and Zhou, 2019; Yang and Park, 2023).

Previous extensive research indicates that various sleep disorders exhibit alterations in global brain network topology, including obstructive sleep apnea (OSA) (Tang et al., 2024; Chen et al., 2018), idiopathic rapid eye movement sleep behavior disorder (Sun et al., 2025), sleep deprivation (Ning et al., 2022; Tian et al., 2024), and poor sleep quality (Ding et al., 2025), characterized by reduced E_glob_, and often altered L_p_, σ, and C_p_. Collectively, these alterations from optimal network organization suggest impaired information processing efficiency and reduced robustness of brain function (Tang et al., 2024; Park et al., 2019). Specifically, the concurrent reductions in E_glob_ and E_loc_ suggest a decline in the brain network’s efficiency for both long-range parallel information transfer and local information processing (Zhu et al., 2020). Furthermore, the decrease in C_p_ indicates a weakening of network functional modularity or segregation, while the reduction in L_p_, in the context of decreased E_glob_ and σ, more likely reflects a shift towards a less optimized, more random-like network structure rather than a simple enhancement of global integration (Sporns, 2018). Crucially, a significant decrease in σ, as observed in our SWSD group and often in other sleep-disrupted states, signifies a deviation of the brain network from the optimal small-world balance that concurrently supports both functional segregation and integration (Zhang et al., 2025). Brain network-level topological changes, stemming from shift work-induced sleep deprivation and circadian misalignment, can disrupt synaptic homeostasis, neuroplasticity, and neural signaling, potentially contributing to associated with cognitive and affective symptoms experienced by individuals with sleep disorders (Ning et al., 2022; Cheong et al., 2023).

Regarding nodal network metrics, our analysis revealed a significant decrease in NCP in the bilateral calcarine cortex, the bilateral lingual gyrus, and the left superior occipital gyrus compared to HCs. Concurrently, NLE was also significantly reduced in this group, specifically in the bilateral calcarine cortex, bilateral lingual gyrus, right cuneus, and right caudate nucleus. These aforementioned visual network regions (bilateral calcarine cortex, bilateral lingual gyrus, left superior occipital gyrus, and right cuneus) are key components of the visual system (Yeo et al., 2011). Their physiological functions range from primary visual perception to higher-order visual cognitive integration, playing an essential role in maintaining vigilance and accurate environmental perception (Dong et al., 2024). The observed reduction in NCP within these visual regions suggests a diminished capacity for these regions to act as connector hubs integrating information from different functional modules (Rubinov and Sporns, 2010). This, in turn, could potentially impair the efficiency of integrating visual information with other cognitive networks, such as the attention and executive control networks (Petersen and Posner, 2012; Seeley et al., 2007; Menon, 2011). Similarly, the significant reduction in NLE within these visual regions indicates impaired information transfer efficiency among their internal neuronal clusters (Achard and Bullmore, 2007), potentially affecting the precision and speed of visual feature extraction (Wang et al., 2010). Furthermore, a significant reduction in NLE was also observed in the right caudate nucleus. As a key component of the basal ganglia, the caudate nucleus plays a pivotal role in cognitive control, learning and memory, reward mechanisms, and motivation regulation (Barrett et al., 2024; Jiang et al., 2023). Impairment of the caudate nucleus may be linked to the impact of sleep deprivation on neuromodulatory systems, such as the dopaminergic system, consequently contributing to the impairments in executive function, decision-making, and motivation maintenance observed in patients with SWSD (Volkow et al., 2012; Gujar et al., 2010; Kreitzer and Malenka, 2008).

### Altered functional network connectivity in SWSD

Beyond alterations in topological properties, this study also revealed a complex subnetwork of altered FC within and between large-scale brain networks (SMN, SN, VN, LN, FPN, DMN), characterized by both abnormally strong and weak connections. In SWSD, abnormally strong connections may reflect the brain’s compensatory efforts to counteract inefficiencies or a dysregulated hyperconnective state, while weakened connections likely indicate reduced information transfer efficiency or impaired integration between regions (Li et al., 2020; Shao et al., 2013). Notably, such compensatory mechanisms are not confined to the brain; similar adaptive responses have been observed at the behavioral level (Bufano et al., 2023), where sleep-deprived individuals exert increased cognitive effort to maintain performance, and in peripheral physiological responses (Cesari et al., 2021), suggesting a system-wide adaptation to the stress of circadian disruption. The VN plays a critical role in processing visual information (particularly light and motion) and sensory modulation (Noseda et al., 2019). A growing body of evidence demonstrates that sleep disorders are associated with significant alterations within key brain networks. Notably, intra-network FC within the VN is consistently disrupted in sleep disorders (Roura et al., 2025). Specific occipital regions, including the precuneus and lingual gyrus, as well as thalamo-cortical visual pathways, show abnormal FC patterns, which have been closely linked to clinical symptoms observed in sleep conditions (Zhang et al., 2013; Wang et al., 2024; Gan et al., 2021). Similarly, the FPN exhibits functional dysregulation characterized by altered internetwork connectivity and activity, which undermines its capacity to support cognitive control processes (Li et al., 2018; Yao et al., 2023). Within the DMN, extensive functional and structural abnormalities are reported across various sleep disorders such as insomnia, obstructive sleep apnea, and sleep deprivation (Zheng et al., 2023; Chang et al., 2020; Wang et al., 2023; Wang et al., 2015). These include aberrant within-network FC, cortical thinning, and regional dysfunction of core DMN nodes (Yu et al., 2018; Marques et al., 2018), coupled with reductions in nodal centrality and local efficiency (Chen et al., 2018; Suh et al., 2016), reflecting diminished information integration and processing within the DMN. Given the DMN’s fundamental role in intrinsic cognition, emotional regulation, and self-referential processing, such intra-network disturbances likely underpin the cognitive deficits and emotional disturbances frequently observed in sleep disorder populations (Huang et al., 2024; McKinnon et al., 2018; Hehr et al., 2023). Furthermore, the limbic system, deeply implicated in emotion regulation and stress response, also shows altered connectivity patterns, with chronic insomnia patients exhibiting abnormal FC between the limbic structures (e.g., hippocampus, amygdala) and reward-related networks (Zhang et al., 2025; Park and Kim, 2023; Gong et al., 2021).

Beyond these intra-network disruptions, sleep disorders also profoundly affect FC between distinct brain networks. These disorders disrupt VN interactions with the DMN (Zhao et al., 2024; Di et al., 2024), and subcortical regions such as the thalamus, where connectivity with the visual cortex is compromised particularly during sleep deprivation (Mai et al., 2022). Functional dysregulation extends to inter-network coupling involving the FPN, which shows altered functional integration with the DMN and VN, thereby weakening executive and attentional control mechanisms (Li et al., 2018; Yao et al., 2023). Additionally, broader network reorganizations involve the VAN and SMN, which display abnormal intra- and inter-network connectivity patterns associated with cognitive-attentional impairments and dysregulation of motor and arousal functions (Ning et al., 2022; Hou et al., 2022; Zhang et al., 2025; Ma et al., 2024). These inter-network connectivity disturbances, combined with the intra-network dysfunctions, provide a comprehensive neural framework for understanding the multifaceted clinical manifestations of sleep disorders.

### Clinical correlates of altered network topology and functional network connectivity

The clinical relevance of these network alterations is underscored by our correlation analyses. First, a negative correlation was observed between PSQI scores and NLE in the right cuneus. The cuneus, a core region of the occipital visual cortex, is involved in primary visual information processing, visuospatial processing, and visual attention (McMains and Kastner, 2011; Vanni et al., 2001). The significant negative correlation we observed between PSQI scores and NLE in the right cuneus therefore implies that poorer sleep quality is associated with lower local information processing efficiency in this region. Previous research has established that sleep deprivation impairs visual attention and visual working memory (Hudson et al., 2020; Abdolalizadeh and Nabavi, 2022). Second, a significant negative correlation was found between ISI scores and FC between the right superior orbital frontal gyrus and the left gyrus rectus. Both regions are key subregions of the orbitofrontal cortex (OFC), a hub for emotion regulation, reward valuation, decision-making, and cognitive flexibility (Rolls et al., 2020). This finding implies that greater insomnia severity in SWSD is accompanied by weaker information exchange within the OFC. Similar patterns of OFC hyper- or hypoconnectivity, which scale with ISI scores, have been reported in primary insomnia cohorts (Li et al., 2017). Given that OFC dysfunction is frequently linked to emotional disorders (e.g., anxiety and depression) and executive function deficits (Rolls et al., 2020; Drevets, 2007), this aberrant FC may relate to the difficulties experienced by patients with SWSD in sleep regulation, emotional control, and cognitive inhibition (Bryden and Roesch, 2015). Our findings further suggest that a decline in the local network function of this core visual processing area may represent a neural basis for such cognitive deficits in SWSD.

### Limitations

Although this study provided valuable insights into the neural underpinnings of SWSD in nurses, there are several limitations that should be considered. First, our study did not incorporate subjective measures to assess daytime sleepiness (e.g., the Epworth sleepiness scale) or circadian chronotype (e.g., the morningness–eveningness questionnaire). These clinical scales would have allowed us to directly correlate behavioral and circadian phenotypes with the observed alterations in brain networks, thereby enhancing the clinical relevance of our findings. Future studies should incorporate these assessments to build a more comprehensive brain-behavior model of SWSD. Second, the cross-sectional design of our study limits causal inference. It remains unclear whether the observed brain changes are a cause or a consequence of SWSD. A longitudinal study would be invaluable, not only to elucidate the direction of causality but also to map the temporal dynamics of these network alterations. Such a design would enable us to determine, for instance, whether these changes are exacerbated by continued shift work, fluctuate with schedule modifications, or can be ameliorated through intervention. Third, the study focused exclusively on female nurses aged 20–40 years, which may limit the generalizability of the findings to other populations, such as male nurses or nurses in different age groups. Importantly, this precludes the exploration of how SWSD-related brain changes may vary across the lifespan. Future studies should include more diverse samples, particularly across a wider age range and in other populations (e.g., male shift workers), to assess the generalizability of these findings. Fourth, the assessment of sleep disorders and psychological status relied on self-report questionnaires (PSQI, ISI, BAI, and BDI-II), which are subject to recall bias and subjective interpretation, although these are standard instruments in the field. Objective measures of sleep, such as actigraphy or polysomnography, could provide more robust data in future research. Fifth, the focus of the present study is on the topological properties and functional network connectivity in nurses with SWSD. However, this approach cannot resolve the directionality of influence between brain regions. To address this limitation, future investigations should employ methods designed to assess effective connectivity, such as Multivariate granger causality. By utilizing a larger sample and multivariate Granger causality analysis, such studies could elucidate the dynamics of directed functional influence and information flow within and between networks, providing a more comprehensive understanding of how SWSD reconfigures the brain’s communication architecture. Sixth, a further limitation is our inability to perform a stratified analysis by shift work duration. Although we collected these data, the resulting subgroups (e.g., 1–5 years, *n* = 10; 6–10 years, *n* = 9; >10 years, *n* = 26) were insufficiently powered for a robust statistical comparison. Future research with larger sample sizes is warranted to elucidate the chronic effects of shift work and, in turn, to better understand the associated neuroplastic changes. Seventh, A significant limitation of this study is the lack of pre-registration prior to data collection. This oversight may have compromised the transparency of the research process. To address this and uphold research integrity, we are committed to rigorously pre-registering all future studies to maximize their rigor and transparency. Finally, our analysis was based on the AAL-90 atlas, which excludes the cerebellum. Recent evidence suggests that the cerebellum is also affected by shift work (Choi et al., 2025). Future studies should incorporate atlases that include the cerebellum to provide a more complete picture of the neural alterations in SWSD.

## Conclusion

In conclusion, this study demonstrates that SWSD in female nurses is characterized by significant alterations in brain functional network topology. Key among these are widespread reductions in both global and nodal network efficiency, particularly within visual processing regions and the caudate nucleus, alongside a complex pattern of disrupted (primarily weakened) FC across multiple brain networks. Crucially, these neuroimaging changes correlated significantly with clinical measures of insomnia severity and sleep quality. Identifying these topological and FC alterations advances our understanding of the pathophysiological mechanisms underlying SWSD and provides novel insights for potential prevention and intervention strategies.

## Data Availability

The raw data supporting the conclusions of this article will be made available by the authors, without undue reservation.
