# Supplementary material for: Altered functional network topology and connectivity in female nurses with shift work sleep disorder
Source: Front Syst Neurosci. 2025 Jul 15;19:1639981. doi: 10.3389/fnsys.2025.1639981 (PMC12303962; doi:10.3389/fnsys.2025.1639981)
Supplement: Supplementary file 1 [file Data_Sheet_1.docx]

**Altered Functional Network Topology and Connectivity in Female Nurses with Shift Work Sleep Disorder**

**Supplementary materials**

**Imaging parameters were as follows**: rs-fMRI: TR/TE = 3000/35ms, 128 volumes, FOV = 24 cm × 24 cm, Slice thickness = 5.0 mm, and voxel size = 3.75 × 3.75 × 4.0 mm; structural 3D-T1: TR = 750 ms, TE = 2.8 ms, FOV = 24 cm × 24 cm, Slice thickness = 1.0 mm, number of slices = 152, flip angle = 15°, and voxel size = 0.5 × 0.5 × 1 mm.

**The preprocessing steps included:** (1) removal of the first 10 time points to allow for signal stabilization; (2) slice-timing correction; (3) realignment for head motion correction (To control for head motion artifacts, frames with a framewise displacement (FD) greater than 0.5 mm were scrubbed. Participants with more than 20% of frames scrubbed were excluded); (4) exclusion of participants with maximum head displacement > 3 mm or absolute rotation > 3°; (5) spatial normalization to the standard MNI space achieved through the DARTEL alignment method; (6) linear regression to reduce errors; (7) regression of nuisance covariates (including Friston-24 head motion parameters, white matter signals, and cerebrospinal fluid signals); and (8) band-pass filtering (0.01-0.1 Hz).

**Total intracranial volume (TIV) extraction**

Brain structural 3D-T1-weighted images were preprocessed using SPM12 and CAT12, including bias field correction, skull stripping, alignment to MNI template, and segmentation into gray matter, white matter, and cerebrospinal fluid. TIV was extracted for all participants.


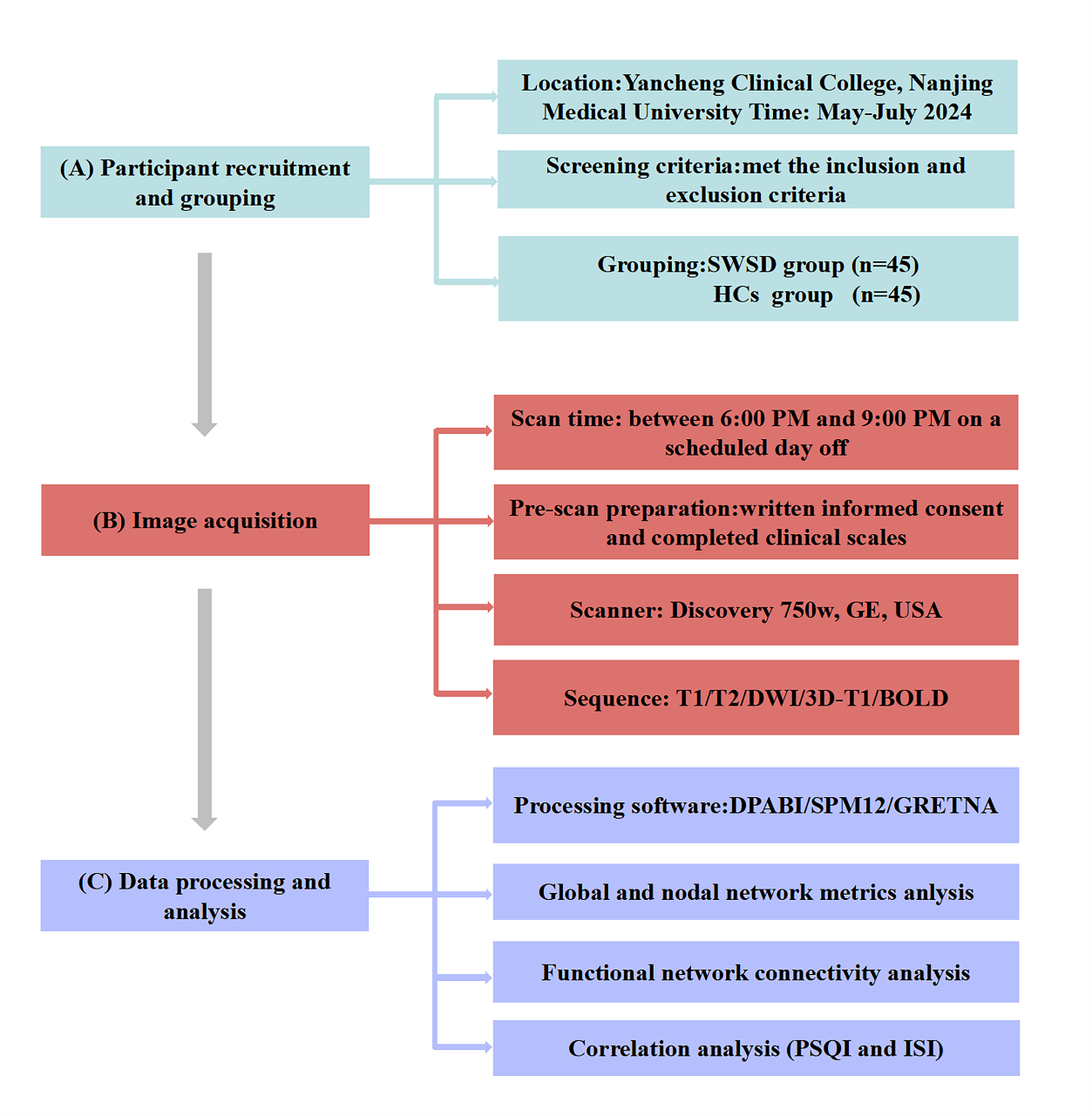


**Figure S1. Flowchart of the neuroimaging study protocol**This methodological framework outlines a three-phase investigation conducted at Yancheng Clinical College (May-July 2024) to analyze brain network alterations and functional connective in female nurses with SWSD. The protocol encompasses: (A) Recruitment of 45 SWSD (PSQI ≥ 5, shift workers) and 45 healthy controls (HCs, PSQI < 5, day-time workers) matched for age/education; (B) Neuroimaging data were acquired on a 3T MRI scanner between 6:00 PM and 9:00 PM. The standardized protocol included T1-weighted, T2-weighted, diffusion (DWI/DTI), and BOLD functional imaging; (C) Data processing using DPABI/SPM12 for preprocessing, AAL-90-based network topology analysis (GRETNA-derived global/nodal metrics), and covariate-adjusted statistical modeling with FDR correction. Subsequently, we conducted brain functional network connectivity analysis and correlation analysis with clinical parameters.

AAL, Automated Anatomical Labeling; BOLD, Blood Oxygen Level Dependent; DPABI, Data Processing & Analysis for Brain Imaging; DTI, Diffusion Tensor Imaging;

DWI, Diffusion-Weighted Imaging; FDR, False Discovery Rate; GE, General Electric;

GRETNA, Graph-theoretical Network Analysis toolbox; HCs, Healthy Controls; ISI, Insomnia Severity Index; MRI, Magnetic Resonance Imaging; PSQI, Pittsburgh Sleep Quality Index; SPM12, Statistical Parametric Mapping (version 12); SWSD, Shift Work Sleep Disorder; USA, The United States of America.
